# Adaptive virtual impedance control strategy based on IWOA-fuzzy PID and its application to reactive power sharing in islanded microgrids

**DOI:** 10.1038/s41598-025-32695-3

**Published:** 2025-12-19

**Authors:** Yongxin Hou

**Affiliations:** School of Electrical and Information Engineering, Tianjin University, Tianjin, 300072 China

**Keywords:** Islanded microgrid, Droop control, Reactive power sharing, Adaptive virtual impedance, Fuzzy PID, Whale Optimization Algorithm, Energy science and technology, Engineering, Mathematics and computing

## Abstract

Accurate reactive power sharing in islanded microgrids is often compromised by resistive line impedances and parameter mismatches, causing power coupling and uneven distribution. This paper proposes an adaptive virtual impedance control strategy that integrates a fuzzy PID controller with the Improved Whale Optimization Algorithm (IWOA). The fuzzy PID ensures nonlinear adaptability, while IWOA globally optimizes fuzzy rules, membership functions, and PID gains for robust self-tuning. By dynamically adjusting virtual resistance and reactance, the strategy reshapes inverter output impedance, decouples active and reactive power, and enables proportional reactive power sharing under diverse network conditions. Simulation studies in MATLAB/Simulink with multiple DG units, varying loads, and unequal capacities verify its effectiveness. Results show improved sharing accuracy, faster response (<0.1 s), and reduced steady-state error compared with conventional droop control, fixed virtual impedance, and PSO-fuzzy-PID methods. The proposed IWOA-fuzzy-PID approach thus offers a practical and scalable solution to enhance stability and reliability in islanded microgrids.

## Introduction

With the rapid growth of renewable energy integration, microgrids have emerged as an effective platform for distributed generation (DG) and are increasingly regarded as a vital component of modern power systems^1,2^. In particular, islanded operation is essential for ensuring reliable electricity supply in remote areas or during main grid outages^3^. However, once disconnected from the utility grid, microgrids face significant challenges in maintaining stability and power quality, with accurate power sharing among parallel DG units being one of the most critical issues^4^.

Droop control is widely used in islanded microgrids due to its decentralized nature and independence from communication links^5^. Conventional droop strategies assume inductive line impedance, enabling decoupled active power frequency (P-f) and reactive power voltage (Q-V) regulation^6^. In practice, however, the low-voltage distribution lines of microgrids often contain considerable resistive components. This leads to strong P-V and Q-f coupling and prevents proportional reactive power sharing^7,8^. Moreover, mismatched line impedances between DGs and the point of common coupling (PCC) further degrade reactive power distribution, potentially overloading certain units and compromising system stability^9^.

To alleviate these problems, the fixed virtual impedance method reshapes the inverter output impedance by introducing constant resistive and reactive terms^10,11^. While effective in reducing coupling, this approach is static and cannot adapt to changing operating conditions, and it often results in unnecessary voltage drops. Adaptive virtual impedance methods based on PID controllers have therefore been explored^12,13^. Although PID regulation provides dynamic adjustment, the gains (Kp, Ki, Kd) are typically tuned offline and remain fixed, limiting robustness under nonlinear and time-varying conditions.

Heuristic optimization methods such as Particle Swarm Optimization (PSO)^14,15^ and Genetic Algorithms (GA)^16,17^ have been applied to improve PID parameter tuning. These methods enhance static performance compared with empirical tuning but still suffer from issues such as premature convergence, local optima, and slow response, restricting their suitability for real-time microgrid control.

In recent years, increasing attention has been given to advanced and AI-based PID control techniques for microgrid applications, aiming to overcome the limitations of conventional PID control under nonlinear and time-varying operating conditions. Several hybrid intelligent controllers have been proposed that integrate machine learning, fuzzy logic, and evolutionary computation into the PID framework to achieve self-tuning and enhanced adaptability.

For example, Neural-Network-PID (NN-PID) and Adaptive Neuro-Fuzzy Inference System (ANFIS)-PID controllers have demonstrated strong nonlinear mapping capability and real-time adaptability, allowing automatic gain adjustment according to load changes and system disturbances^18^. Fuzzy-PID control methods combine human reasoning and mathematical precision to achieve improved steady-state accuracy without relying on a detailed system model^19,20^. Furthermore, deep reinforcement learning (DRL)-based PID and metaheuristic-optimized PID have been successfully applied in islanded AC/DC microgrids to enhance transient response and reactive power regulation^19,21^. However, despite their superior adaptability, these AI-based controllers often involve high computational cost and convergence uncertainty, which limit their real-time implementation in embedded systems.

Considering these factors, the present study integrates fuzzy logic with an Improved Whale Optimization Algorithm (IWOA) to construct a computationally efficient IWOA-Fuzzy-PID controller. The proposed method maintains the interpretability and simplicity of conventional PID control while introducing global optimization and adaptive self-tuning capabilities, thus achieving high performance with reduced parameter-tuning complexity.

To clearly position the proposed method in relation to existing approaches, Table 1 summarizes a comparative analysis of representative PID-based control strategies for islanded microgrids.

**Table 1:** Comparison of PID-based control strategies in islanded microgrids.

Control strategy	Method	Adaptability	Convergence speed	Implementation complexity	Limitations
Conventional PID	Manual tuning	Low	Fast	Low	Poor robustness under varying conditions
Fuzzy-PID	Rule-based reasoning	Moderate	Medium	Medium	Subjective rule design
PSO-Fuzzy-PID	Particle Swarm Optimization	High	Medium	Medium	Prone to local minima; slower convergence
GA-PID	Genetic Algorithm	High	Slow	High	Complex tuning; risk of local optima
ANN/ANFIS-PID	Neural/Neuro-Fuzzy Network	Very high	Variable	High	Requires large training data; computationally heavy
IWOA-Fuzzy-PID	IWOA+Fuzzy Logic	Very high	Fastest ($\sim$0.1s)	Moderate	Balances adaptability, convergence, and efficiency

As summarized in Table 1, the proposed IWOA-Fuzzy-PID achieves a balance between adaptability, convergence speed, and implementation complexity

To address these limitations, this paper proposes an adaptive virtual impedance strategy based on a fuzzy PID controller optimized by the Improved Whale Optimization Algorithm (IWOA). The main contributions are summarized as follows:

1. An adaptive virtual impedance controller with fuzzy PID structure is developed, enabling dynamic adjustment of virtual resistance and reactance for effective power decoupling and accurate reactive power sharing.
2. To overcome the difficulties of fuzzy PID parameter tuning, IWOA is introduced to globally optimize fuzzy rules, membership functions, and initial PID parameters, thereby improving convergence speed and overall control performance.
3. Comprehensive simulations in MATLAB/Simulink under multiple scenarios including multiple DG units, varying loads, and different capacity ratios demonstrate that the proposed method outperforms conventional approaches in terms of reactive power sharing accuracy, dynamic response, and voltage drop reduction.

The remainder of this paper is organized as follows. “Principles of inverter droop control and virtual impedance” section establishes the islanded microgrid model and analyzes the limitations of droop control. “Primary control: IWOA-fuzzy PID adaptive virtual impedance strategy” section details the design of the proposed primary controller (IWOA-fuzzy PID-based adaptive virtual impedance). “Simulation results and analysis” section presents and analyzes the simulation results. Finally, “Conclusions and future work” section concludes the paper and outlines future work.

## Principles of inverter droop control and virtual impedance

### Inverter and its droop control model

**Fig. 1:**
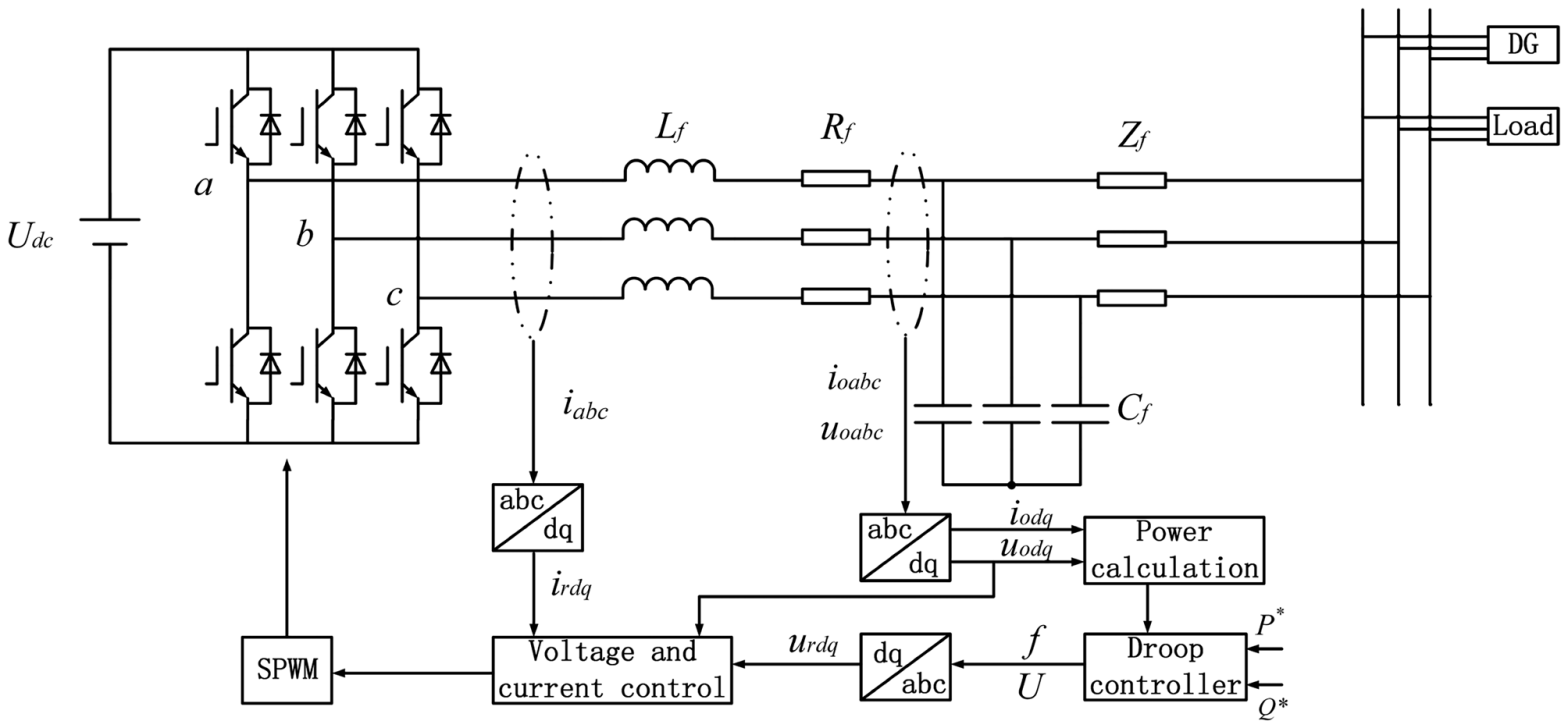
Structure of a three-phase inverter system.

The basic structure of a three-phase inverter system is shown in Fig. 1. The main circuit is supplied by a DC voltage source ${U_{dc}}$ through a three-phase bridge inverter, the phase voltages ${U_{a}}$, ${U_{b}}$, ${U_{c}}$ are generated. The inverter output passes through an LC filter (filter inductor ${L_f}$,filter resistor ${R_f}$, and filter capacitor ${C_f}$ )and is then connected to the point of common coupling (PCC), where the line impedance is denoted as ${Z_{line}}$.

In the control stage, the three-phase voltage and current signals are first transformed from the *abc* stationary frame into the *dq* rotating reference frame. Instantaneous active and reactive power values are calculated based on the transformed signals. According to the measured PQ power, reference values for frequency and voltage are generated via droop equations, and further combined with reference signals ${v_{ref}}$ .These are then processed by the voltage and current control loops, where the voltage loop regulates inverter output voltage and the current loop regulates inverter output current. Finally, the sine pulse width modulation (SPWM) generates the switching signals for inverter operation.

**Fig. 2:**
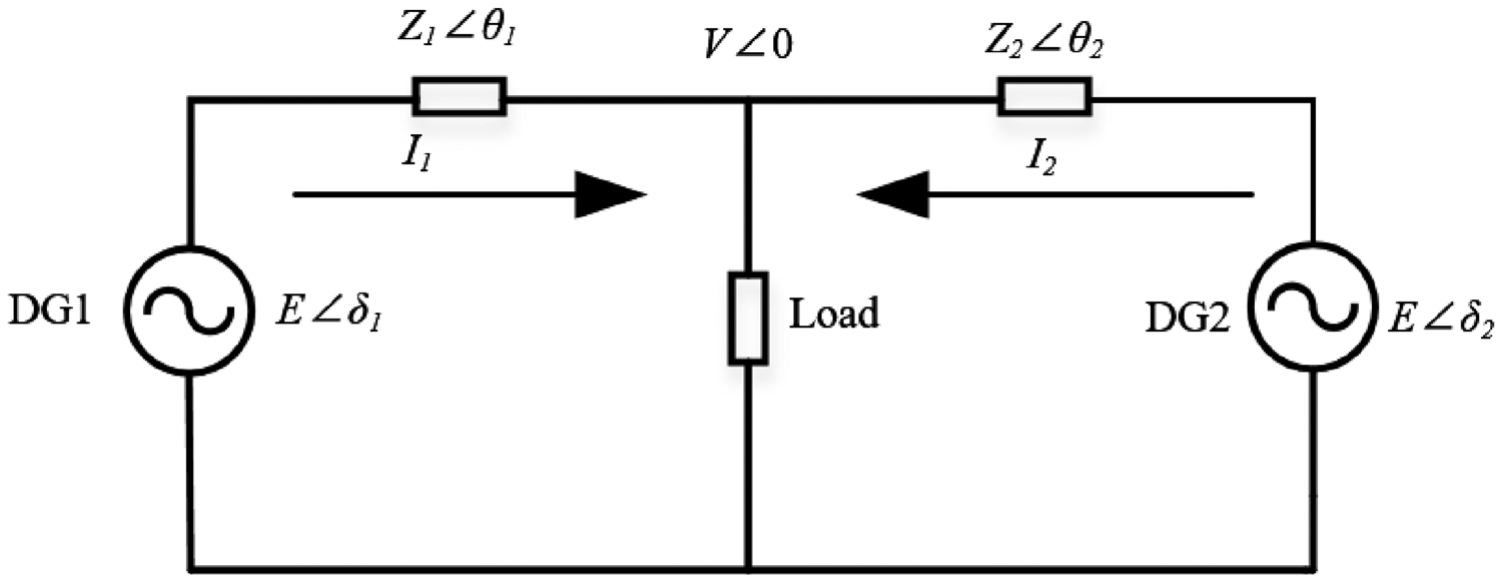
Transmission characteristics of two distributed generation units in parallel.

The droop control model has operating characteristics similar to those of synchronous generators. Its main advantage lies in simplicity and ease of implementation, avoiding the need for centralized control or complex communication links. Conventionally, the droop control laws are expressed as $P - f$ and $Q - V$ , which independently adjust frequency and voltage amplitude based on active and reactive power, respectively. This enables autonomous and decentralized power sharing among multiple units. Figure 2 illustrates the transmission characteristics of two distributed generation (DG) units operating in parallel. Each DG is connected to the point of common coupling (PCC) through an inverter and line impedance. Let the PCC voltage be $V\angle {0^ \circ }$ . The output voltages of the two inverters are denoted as $E\angle {\delta _i}$ , with line impedances represented by $Z\angle {\theta _i} = {R_i} + j{X_i}$ .

According to Ohm’s law, the inverter output current can be expressed as:

1 $$\begin{aligned} \dot{I_i} = \frac{E \angle \delta _i - V \angle 0}{Z \angle \theta _i} \end{aligned}$$

The active and reactive power delivered by the inverter can be derived as :

2 $$\begin{aligned} \left\{ \begin{aligned} P_i&= \frac{1}{Z_i}\Bigl (E_i V \cos \delta _i \cos \theta _i - V^2 \cos \theta _i + E_i V \sin \delta _i \sin \theta _i\Bigr )\\ Q_i&= \frac{1}{Z_i}\Bigl (E_i V \cos \delta _i \sin \theta _i - V^2 \sin \theta _i + E_i V \sin \delta _i \cos \theta _i\Bigr ) \end{aligned} \right. \end{aligned}$$

From the above expressions, it can be seen that the inverter output power depends not only on voltage and phase difference but also on the line impedance *Z* . In high-voltage systems, since the transmission lines are typically long, voltages are high, resistances are small and the line current frequency is relatively low, the line impedance mainly exhibits inductive characteristics. When $\theta \rightarrow {90^ \circ }$ and $\delta \rightarrow 0$ , one can approximate $\sin \delta = \delta$ and $\cos \delta = 1$. Equation (2) can then be simplified to (3):

3 $$\begin{aligned} \left\{ \begin{array}{l} P_i = \frac{E_i V}{X_i} \delta _i \\ Q_i = \frac{E_i V - V^2}{X_i} \end{array} \right. \end{aligned}$$

From (3), it can be seen that when the line impedance exhibits inductive characteristics, the active power of the system is mainly linearly related to the phase angle variation, whereas the reactive power is approximately linearly related to the voltage amplitude variation. This feature lays the theoretical foundation for droop control. Although certain parameter deviations exist in practice, this approximate linear relationship is still applicable. Therefore, when the transmission line impedance can be regarded as predominantly inductive, the droop control law can be expressed as:

4 $$\begin{aligned} {\left\{ \begin{array}{ll} f_i = f^{*} - m_i P_i \\ E_i = E^{*} - n_i Q_i \end{array}\right. } \end{aligned}$$

**Fig. 3:**
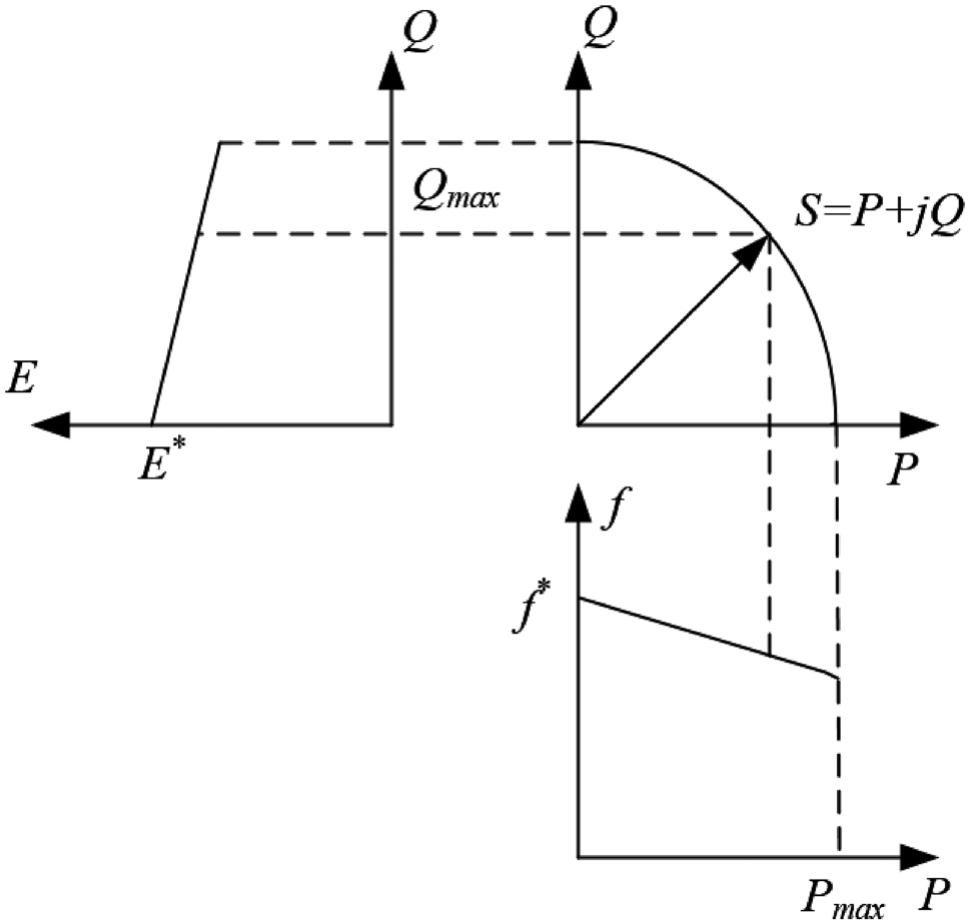
Droop curve with inductive line characteristics.

The droop control characteristics are shown in Fig. 3, where *f* is the actual output frequency of the inverter, ${f^ * }$ is the reference frequency, *E* is the actual output voltage amplitude of the inverter, ${E^ * }$ is the reference voltage amplitude, and *m* and *n* are the active and reactive power droop coefficients, respectively.

As shown in the figure, the system frequency has a linear relationship with active power, while the inverter output voltage amplitude has an approximately linear relationship with reactive power. Based on this active power frequency and reactive power voltage linear relationship, droop control enables effective autonomous parallel operation of multiple DG units without communication, thereby achieving rational power sharing among units according to their capacities and improving the reliability of microgrid operation.

### Impact of resistive lines on droop control

In islanded microgrids, frequency is a global variable. By adjusting the $P - f$ droop coefficient in (4), active power sharing can be achieved among multiple DG units.

Similarly, reactive power sharing is realized through $Q - V$ droop control. However, traditional droop control is established under the assumption of inductive line impedance. In practice, the output lines of low-voltage microgrids contain non-negligible resistive components, which cause deviations in active power sharing and hinder accurate proportional distribution according to DG capacities.

When the line impedance is dominantly resistive $R> > X$, according to (2), the relationship between the DG output power and the PCC voltage can be simplified as:

5 $$\begin{aligned} \left\{ \begin{array}{l} P_i = \dfrac{E_i V - V^2}{R_i} \\ Q_i = - \dfrac{E_i V}{R_i} \delta _i \end{array} \right. \end{aligned}$$

From (5), it is evident that active power is mainly related to the voltage amplitude, while reactive power is correlated with the phase angle (frequency). This contradicts the assumptions of conventional droop control and leads to severe coupling between active and reactive power.

Moreover, as shown in Fig. 2, if the DGs have identical power ratings but different line impedances, mismatches in line resistance will further aggravate the imbalance. For instance, when the line impedance of DG1 is smaller than that of DG2, DG1 will bear a larger share of reactive power.

The line voltage drop can be expressed as (6):

6 $$\begin{aligned} {\left\{ \begin{array}{ll} \Delta U_1 \approx \dfrac{X_1 Q_1 + R_1 P_1}{U_0} \\ \Delta U_2 \approx \dfrac{X_2 Q_2 + R_2 P_2}{U_0} \end{array}\right. } \end{aligned}$$

In the equation, $\Delta {U_1}$ and $\Delta {U_2}$ represent the voltage drops from the distributed power sources to the common coupling point. When the line impedance is inductive, the equations simplify as:

7 $$\begin{aligned} \left\{ \begin{array}{l} \Delta U_1 \approx \dfrac{X_1 Q_1}{U_0} \\ \Delta U_2 \approx \dfrac{X_2 Q_2}{U_0} \end{array} \right. \end{aligned}$$

The total voltage at the PCC is then given by:

8 $$\begin{aligned} \left\{ \begin{array}{l} {U_1} = \Delta {U_1} + {U_{PCC}}\\ {U_2} = \Delta {U_2} + {U_{PCC}} \end{array} \right. \end{aligned}$$

**Fig. 4:**
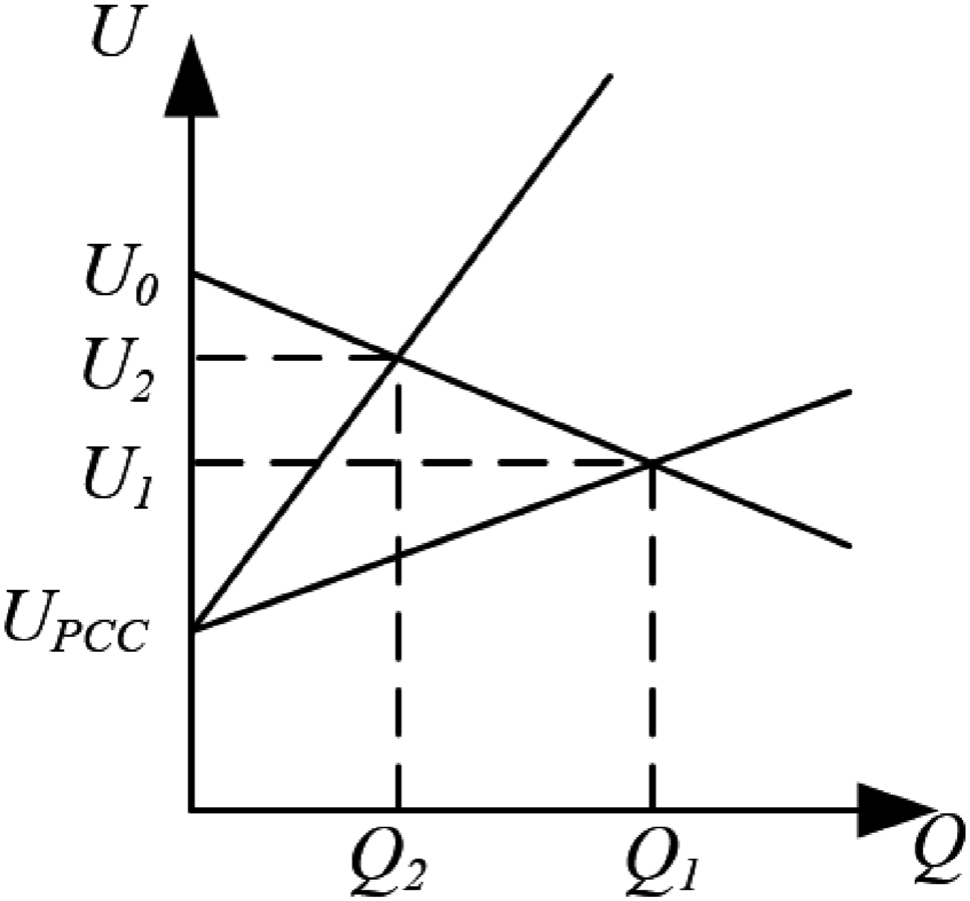
Reactive power sharing of DGs.

As shown in Fig. 4, the output power of DG1 and DG2 shows a noticeable phase shift, and the slope of the corresponding droop curves is greater. This is due to the line impedance being inductive for both sources. When the voltage at both sources remains the same, the reactive power sharing will not achieve proportional power sharing between DG1 and DG2, resulting in inefficient distribution.

In summary, traditional droop control in resistive islanded microgrids suffers from two inherent limitations:

1. Power coupling: the strong $P - V$ and $Q - f$ coupling undermines the decoupling characteristics of conventional droop control.
2. Reactive power sharing: mismatched line impedances prevent accurate capacity-proportional sharing of reactive power.

To address these issues, the virtual impedance method has emerged as an effective improvement. However, its development still faces several challenges, summarized as follows:

1. Fixed virtual impedance: This is the simplest and most direct approach, which reshapes output impedance by embedding constant resistive and reactive components . Nevertheless, it is essentially a static compensation strategy. It cannot adapt to dynamic load variations or operational differences among DG units, and it introduces additional voltage drops that limit optimization effectiveness.
2. Adaptive virtual impedance with conventional PID: To enhance adaptability, PID controllers have been employed to dynamically adjust virtual impedance. Although this alleviates the rigidity of fixed impedance, the PID gains (${k_{p}}$, ${k_{i}}$, and ${k_{d}}$) are typically determined empirically and remain unchanged. Given the nonlinear and time-varying nature of microgrids, fixed-parameter PID controllers cannot guarantee optimal performance across all operating conditions, leading to insufficient robustness.
3. Integration with conventional optimization algorithms: To overcome the PID tuning problem, researchers have applied Particle Swarm Optimization (PSO), Genetic Algorithms (GA), and similar approaches for offline or online optimization of PID gains or virtual impedance. While these methods can improve static performance compared with empirical tuning, classical algorithms such as standard PSO and GA suffer from inherent drawbacks?premature convergence, local optima, and slow convergence?that restrict their dynamic regulation capability.

Existing methods are limited either in adaptability, control accuracy and robustness, or optimization efficiency and real-time applicability. Therefore, a novel adaptive strategy is urgently required. To this end, this paper proposes an integrated solution that combines the IWOA, fuzzy logic, and PID control:

1. Fuzzy logic is employed to handle system nonlinearities and uncertainties through rule-based intelligent adjustment.
2. PID control is retained to ensure steady-state accuracy and eliminate static errors.
3. IWOA is introduced to globally optimize fuzzy rules, membership functions, and initial PID gains, thereby overcoming the deficiencies of conventional optimization algorithms and ensuring faster convergence and superior overall performance.

The proposed strategy enables online self-tuning and self-optimization of virtual impedance, thereby achieving precise power decoupling and reactive power sharing under diverse operating conditions. The design principle and implementation details are elaborated in the following sections.

## Primary control: IWOA-fuzzy PID adaptive virtual impedance strategy

### Virtual impedance principle

**Fig. 5:**
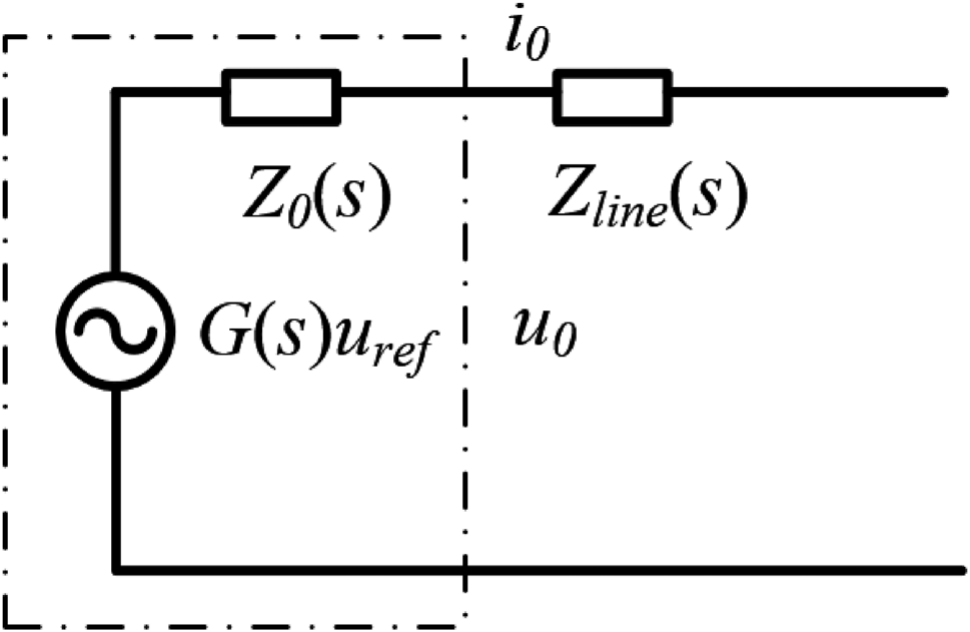
Equivalent circuit under practical conditions for the inverter.

In microgrids, the nodes are interconnected by transmission lines, and the power transfer and distribution are affected by line impedance. Inverters in the microgrid must control the output power according to the demand and supply of electricity in the grid while ensuring stable operation. However, since line impedance is influenced by factors such as path, length, and environmental conditions, its resistance and inductance are difficult to measure accurately. As a result, the inverter output power often fails to match the rated capacity. To address this, virtual impedance is introduced to assist with power control in the microgrid. The equivalent circuit diagram under real conditions is shown in Fig. 5.

The output voltage of the inverter is given by:

9 $$\begin{aligned} {u_0}(s) = G(s){u_{ref}}(s) - {Z_0}(s){i_0}(s) \end{aligned}$$

The output voltage of the inverter after the introduction of virtual impedance is:

10 $$\begin{aligned} {u_0}(s) = G(s){u_{ref}}(s) - [{Z_v}(s)k + {Z_0}(s)]{i_0}(s) \end{aligned}$$

Thus, after the introduction of virtual impedance, the total output impedance of the inverter is calculated as:

11 $$\begin{aligned} Z_0^\prime(s) = G(s) Z_v(s) + Z_0(s) \end{aligned}$$

**Fig. 6:**
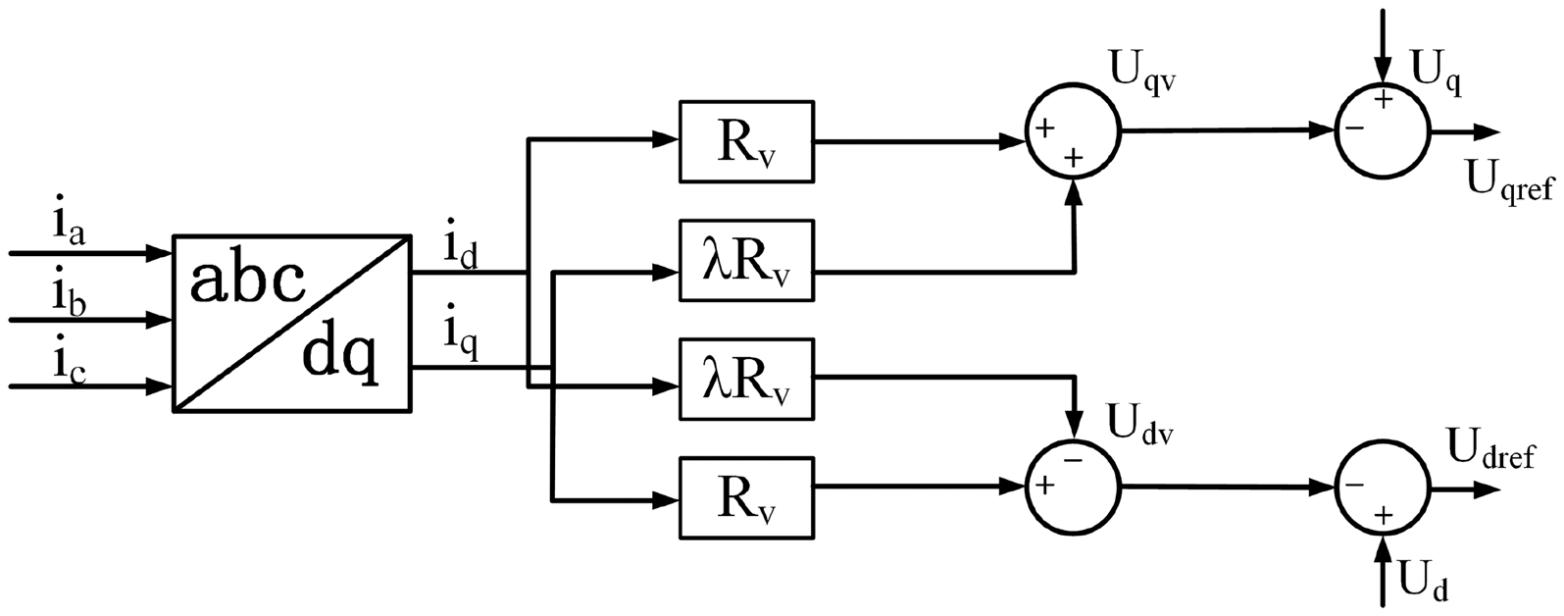
Control structure of virtual impedance.

Figure 6 shows the control structure of virtual impedance in the inverter double-loop system.

### Fuzzy PID adaptive regulator

Fixed-value virtual impedance cannot adapt to load variations or line parameter differences. Therefore, an intelligent method is required to automatically adjust ${R_v}$ and ${X_v}$ according to system states. Fuzzy control provides effective regulation without requiring an accurate mathematical model of the controlled object, but its performance in eliminating steady-state errors is relatively limited. In contrast, PID control offers precise adjustment based on measured error data, particularly in steady-state regulation, though it has limited adaptability to nonlinear and time-varying systems. By combining the strengths of both approaches, a fuzzy PID control strategy is adopted in this paper.

Here, fuzzy logic is used to enhance PID control. The input variables of the fuzzy controller are defined as the reactive power deviation $\Delta Q$ and its first-order derivative $\Delta {Q_C} = d(\Delta Q)/dt$ . The initial parameters of the PID controller, $k_p(0)$, $k_i(0)$, and $k_d(0)$, are set accordingly, and the fuzzy output is fed into the PID regulator.

**Fig. 7:**
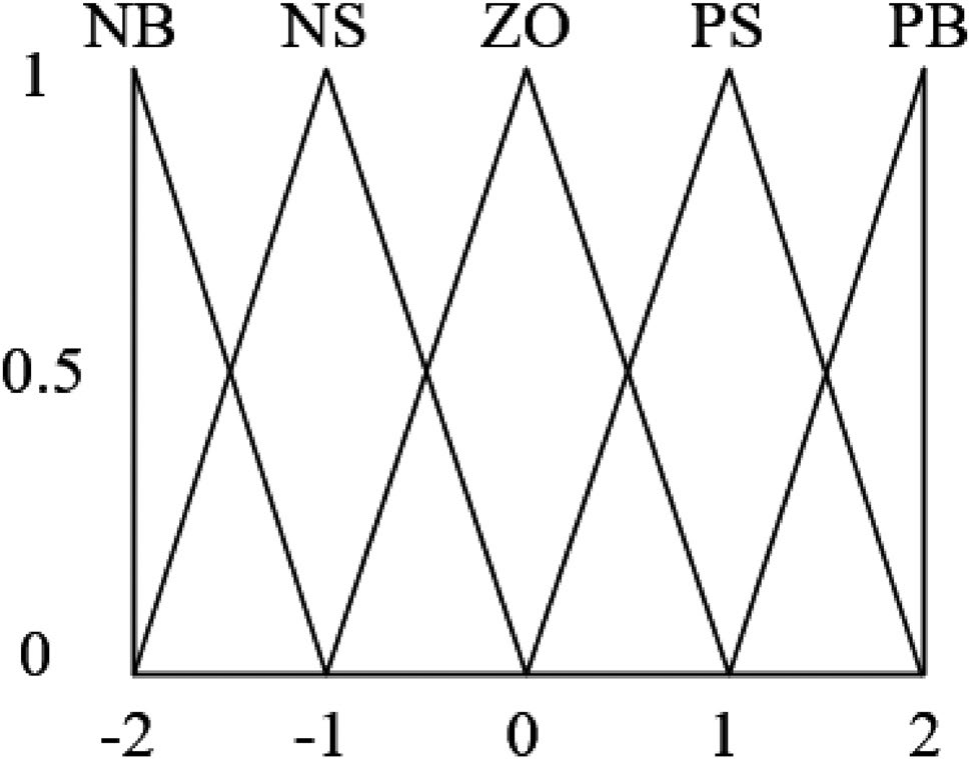
Membership functions of the output variables.

The universes of discourse for the fuzzy inputs are normalized to the range [− 2, 2]. Five fuzzy sets are defined: NB, NS, ZO, PS, and PB, representing negative big, negative small, zero, positive small, and positive big, respectively. For each input variable, the membership functions are constructed as triangular functions, as shown in Fig. 7. Similarly, the output variables adopt triangular membership functions, with the details provided in Tables 2, 3, and 4.

**Table 2:** Fuzzy rule inference table for $k_p$.

$\Delta {Q_\mathrm{{c}}}/\Delta Q$	NB	NS	ZO	PS	PB
NB	PB	PS	PS	PS	ZO
NS	PS	PS	PS	ZO	NS
ZO	PS	PS	ZO	NS	NS
PS	PS	ZO	NS	NS	NS
PB	ZO	NS	NS	NS	NB

**Table 3:** Fuzzy rule inference table for $k_i$.

$\Delta Q_c / \Delta Q$	NB	NS	ZO	PS	PB
NB	NS	NB	NB	NB	PS
NS	ZO	NS	NS	NS	ZO
ZO	ZO	NS	ZO	PS	ZO
PS	ZO	ZO	PS	PS	PB
PB	ZO	NB	NB	NB	ZO

**Table 4:** Fuzzy rule inference table for $k_d$.

$\Delta Q_c / \Delta Q$	NB	NS	ZO	PS	PB
NB	NB	NS	ZO	PS	ZO
NS	NS	NS	ZO	PS	PS
ZO	NS	ZO	PS	PS	PS
PS	ZO	ZO	PS	PS	PB
PB	ZO	PS	PS	PS	PB

Since the design of fuzzy rules and membership functions solely relies on expert knowledge, it may suffer from subjectivity. To address this issue, the IWOA is employed to optimize the initial parameters and membership functions of the fuzzy PID controller, thereby enhancing the convergence speed and accuracy of the controller.

### Improved Whale Optimization Algorithm (IWOA) optimization

#### Standard Whale Optimization Algorithm

The Whale Optimization Algorithm (WOA) is a metaheuristic optimization method inspired by the hunting behavior of humpback whales, first proposed by Mirjalili et al. Compared with Genetic Algorithms (GA) and Particle Swarm Optimization (PSO), WOA features a simple structure, few control parameters, and ease of implementation, while effectively balancing global exploration and local exploitation. The algorithm updates positions through three mechanisms encircling prey, bubble-net attacking, and random search achieving good convergence performance and strong resistance to premature convergence.

1. Encircling prey: The search domain of whales corresponds to the global solution space. To encircle the prey, the location of the prey must be determined first. Since the exact position of the prey is unknown, the current best solution is assumed to represent the prey’s location. Other whales gradually move toward this best solution to complete the encirclement, with the position update described by Eqs. (12) and (13).
  12 $$\begin{aligned} & x\left( t + 1 \right) = x^{*}\left( t \right) - A \times D \end{aligned}$$
  13 $$\begin{aligned} & D = \left| {C \times {x^*}\left( t \right) - x\left( t \right) } \right| \end{aligned}$$
  where *t* denotes the current iteration$t \in \left[ 1, t^{\max }\right]$; ${x^*}\left( t \right)$ is the current best solution; *D* represents the distance between the search agent and the best solution; $x\left( t \right)$ is the current position of the search agent; *A* and *C* are coefficient vectors defined as $A = 2a \times r - a$, $C = 2 \times r$ where *a* is the convergence factor that decreases linearly from 2 to 0, and is a random number within the interval [0,1]. Accordingly, the variation range of the coefficient vector *A* is $\left[ { - a,a} \right]$.
2. Bubble-net attacking:The bubble-net feeding behavior of humpback whales mainly consists of two mechanisms: shrinking encirclement and spiral updating. When bubble-net attacking is adopted, the position of a whale relative to the prey is updated according to a logarithmic spiral, as expressed in (14) and (15):
  14 $$\begin{aligned} & x\left( t + 1 \right) = D' \times \textrm{e}^{bl} \times \cos \left( 2\pi l \right) + x^{*}\left( t \right) \end{aligned}$$
  15 $$\begin{aligned} & D' = \left| x^{*}\left( t \right) - x\left( t \right) \right| \end{aligned}$$
  where $D'$ denotes the distance between the current search agent and the best solution, *l* is a random number uniformly distributed in [−1,1], *b* is a constant defining the spiral shape. Since the hunting process alternates between the two mechanisms, WOA selects either the spiral updating position or the shrinking encirclement position according to a probability , *p* as given in (16).
  16 $$\begin{aligned} x\left( t + 1 \right) = {\left\{ \begin{array}{ll} x^{*}\left( t \right) - A \times D, & p < 0.5 \\ D' \times \textrm{e}^{bl} \times \cos \left( 2\pi l \right) + x^{*}\left( t \right) , & p \ge 0.5 \end{array}\right. } \end{aligned}$$
  As the number of iterations increases, the parameters *A* and the convergence factor *a* gradually decrease. When $\left| A \right| < 1$ , whales move toward the best solution, and WOA performs local exploitation.
3. Searching for prey: To ensure that whales can explore the solution space sufficiently, WOA updates the position of search agents based on the distance to randomly selected individuals, thereby achieving global exploration. Specifically, when $\left| A \right| \ge 1$ , the search agents move randomly according to:
  17 $$\begin{aligned} & x\left( t + 1 \right) = x_\mathrm{{rand}}\left( t \right) - A \times D'' \end{aligned}$$
  18 $$\begin{aligned} & D'' = \left| C \times x_\mathrm{{rand}}\left( t \right) - x\left( t \right) \right| \end{aligned}$$
  where $D'$ represents the distance between the search agent and a randomly chosen individual, and ${x_{\mathrm{{rand}}}}\left( t \right)$ is the position of a randomly selected whale at iteration *t* .

#### Improved Whale Optimization Algorithm

1. Nonlinear convergence factor:In the standard WOA, the main control parameter vector balances local exploitation and global exploration. As discussed above, $\left| A \right|$ is primarily determined by the convergence factor a, which decreases linearly with iterations. Therefore, the adjustment of a plays a critical role. In multi-objective optimization problems with a large number of dimensions and iterations, the linear decline of a may cause WOA to prematurely enter local exploitation in the mid-to-late stages of the search, thereby weakening its global search capability and reducing optimization performance. To address this, a is increased during the early iterations to strengthen the global exploration ability of WOA, and decreased during the later iterations to refine local exploitation. This adaptive nonlinear strategy modifies the convergence factor according to the following piecewise function (19):
  19 $$\begin{aligned} a\left( \varepsilon \right) = {\left\{ \begin{array}{ll} 2 - \left( \frac{\varepsilon }{M} \right) ^2, & \varepsilon \le \frac{M}{2} \\ 1 - \frac{2\left( \varepsilon - \frac{\varepsilon }{M} \right) }{M} + \left( \frac{\varepsilon - \frac{\varepsilon }{M}}{M} \right) ^2, & \varepsilon > \frac{M}{2} \end{array}\right. } \end{aligned}$$
  where *M* denotes the maximum number of iterations.
2. Diversity mutation operation: To prevent the population from converging prematurely to one or several specific positions, which increases the risk of falling into local optima, a biological concept of species aggregation degree is introduced. The aggregation index 1/*k* is defined as follows:
  20 $$\begin{aligned} \frac{1}{k} = \frac{V - m}{m^2} \end{aligned}$$
  where *V* denotes the sum of species fitness values, and *m* is the average fitness of the population. When $1/k \ge 0$ , the population exhibits an aggregation state; when $k \rightarrow \infty$ , the population behaves randomly. To avoid premature aggregation in the early search stage, during the range $\varepsilon \le \textrm{M}/2$ , $1/k = 0.125$ is set as the threshold to trigger mutation operations. The mutation formula is defined as:
  21 $$\begin{aligned} X\left( \varepsilon + 1 \right) = X\left( \varepsilon \right) \left( 1 + 0.5\xi \right) \end{aligned}$$
  where $\xi$ is a random variable obeying a normal distribution.
3. Fitness Function Definition In the proposed PID parameter optimization process, the Improved Whale Optimization Algorithm (IWOA) evaluates the performance of each candidate solution $\textbf{X} = [K_p, K_i, K_d]$ by computing a cumulative control error through the discrete-time system response. The fitness function corresponds to the total cost *F*, which is minimized during the optimization. For each iteration step *k*, the instantaneous control error is defined as $e_k = r_k - y_k$, where $r_k$ and $y_k$ denote the reference input and the system output, respectively, and $u_k$ is the control signal. The discrete objective function used in this study can be expressed as:
  22 $$\begin{aligned} F = \sum _{k=1}^{P} \left( 0.999|e_k| + 0.001u_k^2 \right) + \sum _{k=2}^{P} 100|\Delta y_k| \cdot \textbf{1}\{\Delta y_k < 0\}, \end{aligned}$$
  where $\Delta y_k = y_k - y_{k-1}$, and $\textbf{1}\{\cdot \}$ is an indicator function that equals 1 when the condition in braces is satisfied and 0 otherwise. The first term penalizes the absolute tracking error and excessive control effort, while the second term imposes an additional penalty when the system output decreases, thereby suppressing oscillations and ensuring smooth convergence. The total accumulated value of *F* (denoted as BsJ in the simulation code) is taken as the fitness value in the optimization process, and the IWOA algorithm iteratively minimizes *F* to obtain the optimal PID parameters.

**Fig. 8:**
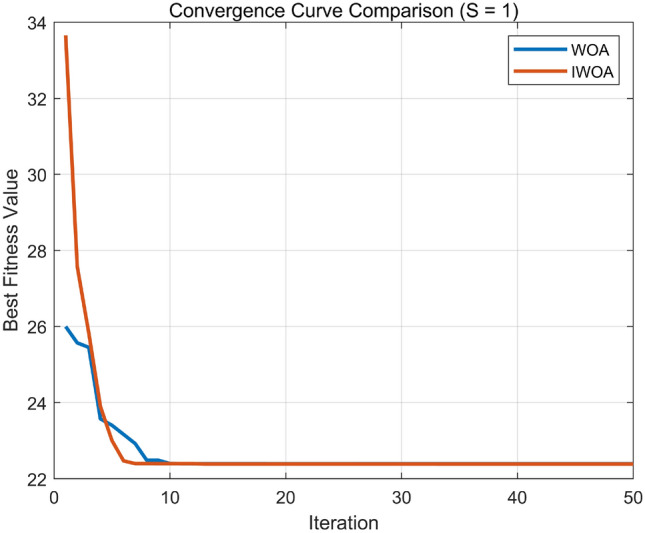
Convergence curves of WOA and IWOA under step input.

**Fig. 9:**
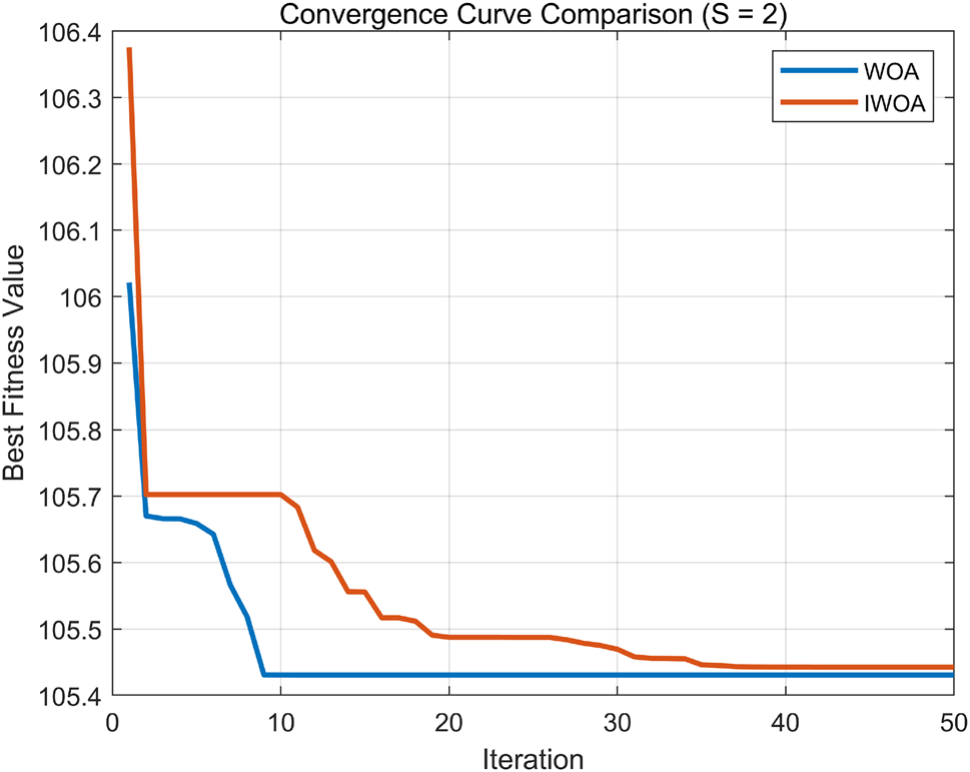
Convergence curves of WOA and IWOA under sinusoidal input.

To further verify the effectiveness of the Improved Whale Optimization Algorithm (IWOA), comparative simulations were carried out between the standard WOA and the proposed IWOA under identical parameter settings. Two types of input signals were used: a unit step signal and a sinusoidal tracking signal. The results of convergence performance and time-domain response are shown in Figs. 8 and 9, respectively. The general parameter configuration for both algorithms is as follows:

- Number of search agents: $SearchAgents\_no = 50$
- Maximum number of iterations: $Max\_iter = 50$
- Decision dimension: $d = 3$ (corresponding to $[K_p, K_i, K_d]$)
- Variable bounds: $$\textbf{lb} = [0,\,0,\,0]$$, $$\textbf{ub} = [50,\,50,\,5]$$

As shown in the convergence curves, both algorithms can rapidly converge under step input conditions, indicating that for relatively simple and single-peak objective functions, the basic WOA already achieves satisfactory optimization performance. However, for sinusoidal inputs with stronger nonlinearity and more complex error surfaces, the standard WOA exhibits premature convergence, while the IWOA continues to improve in later iterations owing to its nonlinear convergence factor and adaptive diversity mutation mechanism. Consequently, IWOA achieves a lower final fitness value, demonstrating better global exploration and robustness.

The corresponding time-domain responses further confirm this observation. Under the step signal, the responses of both WOA-PID and IWOA-PID controllers show similar dynamic characteristics with short rise time and small overshoot. In contrast, under sinusoidal input, the IWOA-PID controller provides smaller steady-state error and reduced oscillation, maintaining a more accurate tracking performance compared with the WOA-PID controller.

Overall, these comparative results validate that the IWOA enhances the balance between global search and local exploitation. It effectively mitigates early stagnation, accelerates convergence in later iterations, and improves optimization precision.

**Fig. 10:**
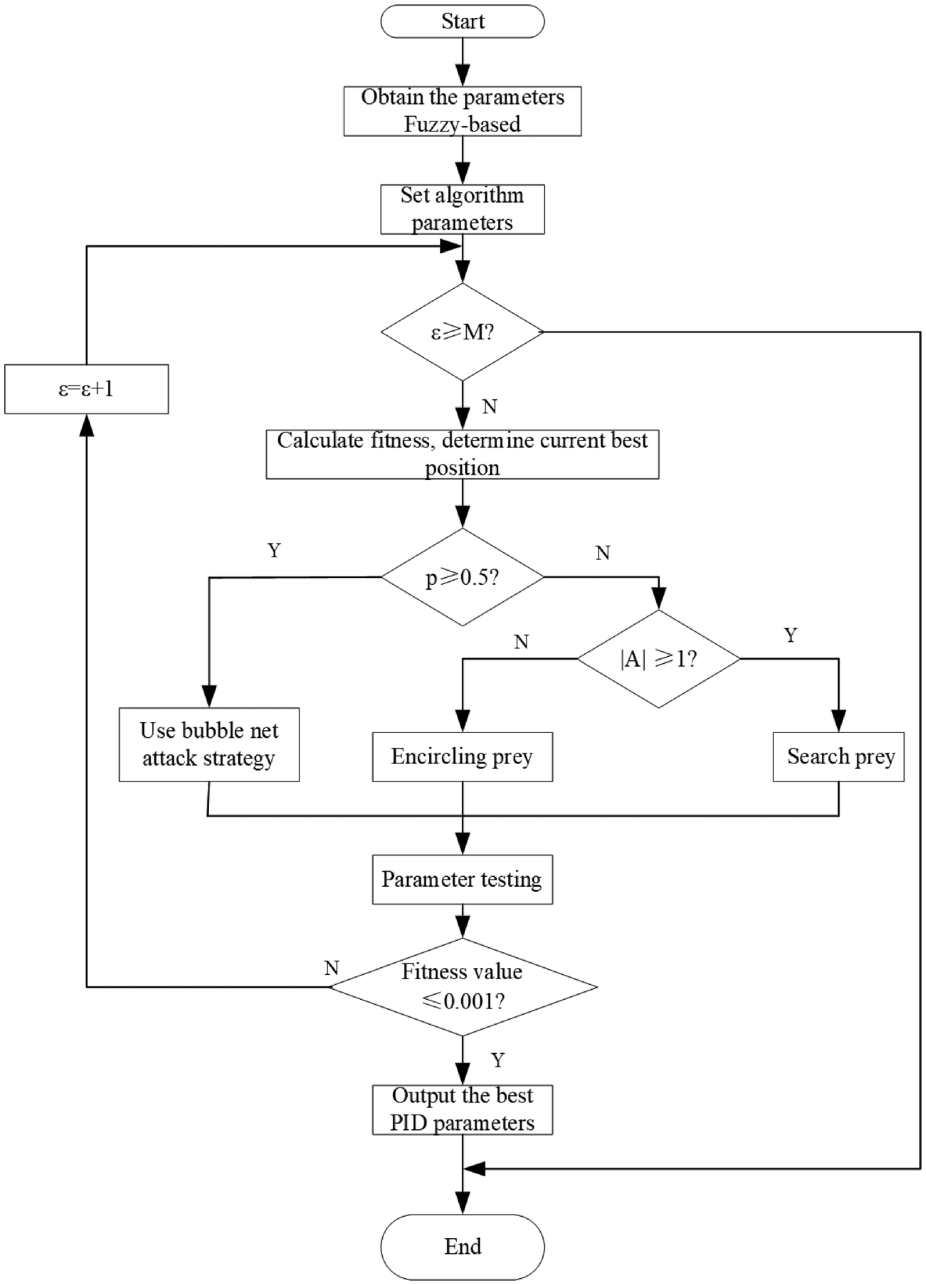
Flowchart of the IWOA.

The flow of the IWOA is illustrated in Fig. 10.

### Overall control structure

**Fig. 11:**
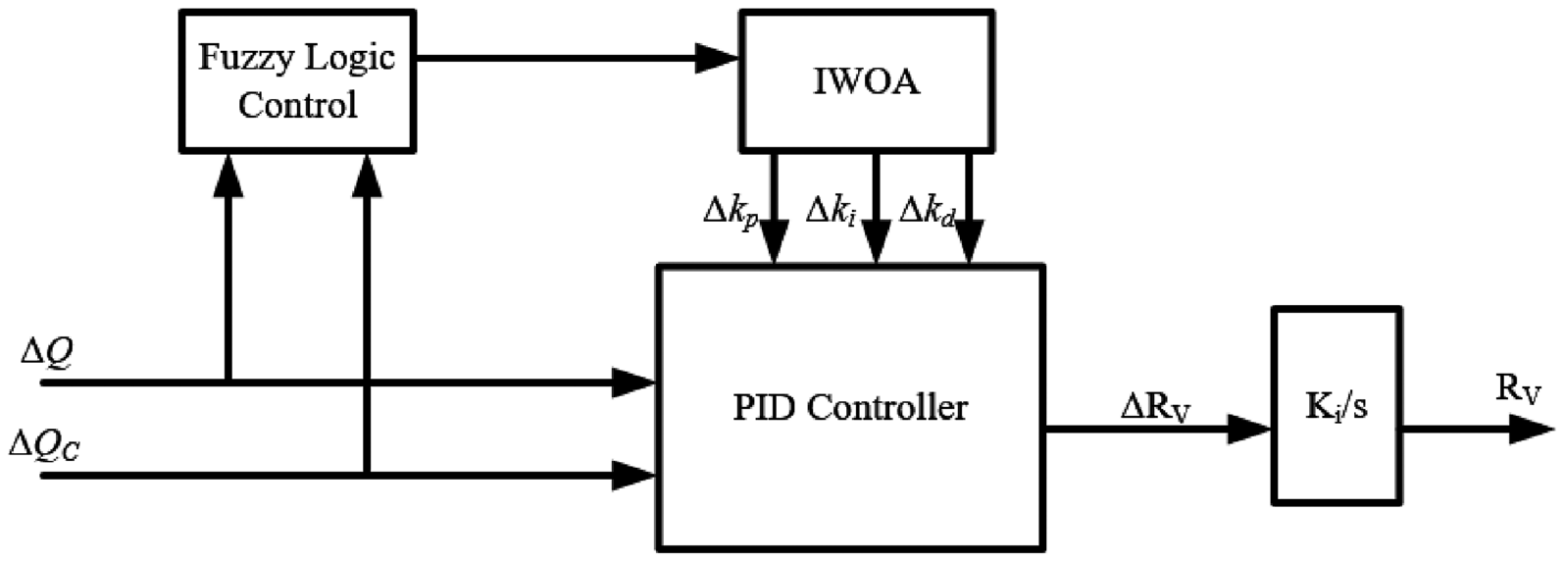
Control structure of adaptive virtual impedance.

Based on the above analysis, the proposed improved primary control strategy is employed to achieve accurate control of reactive power sharing. The overall control block diagram is shown in Fig. 11.

From (6), it can be obtained that:

23 $$\begin{aligned} \begin{aligned} \Delta U_2&\approx \frac{X_2 Q_2 + R_2 P_2}{U_0} \\&= \frac{X_1 Q_2 + R_1 P_2}{U_0} + \frac{\Delta X Q_2 + \Delta R P_2}{U_0} \\&= \Delta U_2^\prime + \Delta U_2'' \end{aligned} \end{aligned}$$

where $\Delta U_2$ denotes the total voltage drop across the line impedance of DG2. It can be decomposed into two parts: the equivalent component $\Delta U_2^\prime$ and the nonequivalent component $\Delta U_2''$ . Therefore,an adaptive virtual impedance loop is introduced to ensure that the equivalent impedance values of multiple lines are equal, thereby eliminating the influence of mismatch.

If the virtual impedance applied to DG2 is defined as $Z_v = R_v + j X_v$ , where $R_v$ and $X_v$ represent the virtual resistance and reactance, respectively, then the voltage drop generated by $Z_v$ satisfies:

24 $$\begin{aligned} \Delta {U_v} + \Delta U_2^{''} = 0 \end{aligned}$$

Substituting into the voltage drop equation yields:

25 $$\begin{aligned} \frac{{X_v Q_2 + R_v P_2}}{{U_0}} + \frac{{\Delta X Q_2 + \Delta R P_2}}{{U_0}} = 0 \end{aligned}$$

In practice, due to uncertain resistive-inductive characteristics of low-voltage lines, direct measurement of $\Delta R$ and $\Delta X$ is challenging. Assuming ${X_v} = {R_v}$ provides a balanced adjustment of resistive and reactive compensation, ensuring that both P-V and Q-f couplings are mitigated simultaneously. This equivalence simplifies computation and has been verified through sensitivity analysis to maintain voltage stability with less than 2% deviation.

26 $$\begin{aligned} R_v = - \frac{\Delta R + \Delta X \cot \varphi }{1 + \cot \varphi } \end{aligned}$$

Where $\cot \varphi = {P_2}/{Q_2}$ . For any given ${P_2}$ , ${Q_2}$ ,$\Delta X$ and $\Delta R$ , there always exists a corresponding value of ${R_v}$ that satisfies (25). When remains constant, the power factor can be maintained. Once $\cot \varphi$ varies, the virtual resistance must be adaptively adjusted. By properly regulating the virtual resistance, the variation of the power factor caused by control parameter changes can be compensated, thus ensuring proportional power sharing and enhancing the overall operational performance of the system. In Fig. 1, the three-phase currents ${i_a}$ ,${i_b}$ , ${i_c}$ are transformed by Park transformation into the synchronous reference frame, yielding the direct-axis and quadrature-axis components ${i_d}$ and ${i_q}$ . By multiplying these with the virtual impedance, the virtual voltage drops on the *d* and *q* , ${u_{dv}}$ and ${u_{qv}}$ , are obtained. The reference output voltage in the synchronous frame can thus be expressed as:

27 $$\begin{aligned} \left( \begin{array}{c} U_{dv} \\ U_{qv} \end{array} \right) = \left( \begin{array}{cc} - \lambda R_v & R_v \\ R_v & \lambda R_v \end{array} \right) \left( \begin{array}{c} i_d \\ i_q \end{array} \right) \end{aligned}$$

**Fig. 12:**
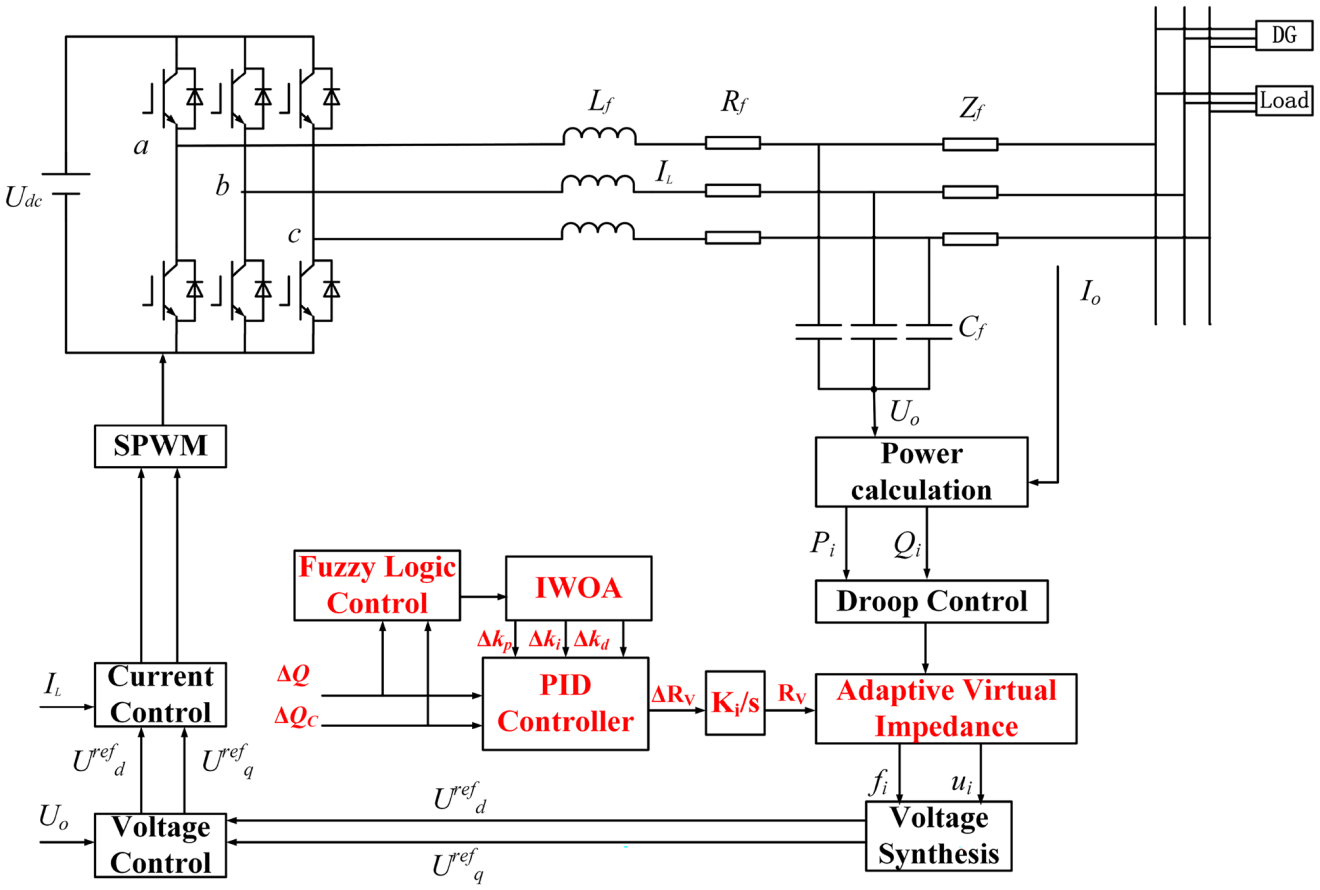
Primary control structure of the islanded microgrid.

As shown in Fig. 12, by introducing an adaptive virtual impedance loop, the effective line impedance can be equalized, thereby realizing accurate reactive power sharing. This design ensures flexibility and robustness under varying system parameters, while avoiding static tuning deficiencies. With the aid of fuzzy PID control optimized by IWOA, the virtual impedance value can be adaptively adjusted online to ensure proportional reactive power sharing and maintain precise power factor regulation, forming the complete primary control strategy.

## Simulation results and analysis

### Simulation platform and parameter settings

In this section, MATLAB/Simulink is employed as the simulation platform to verify the effectiveness of the proposed control strategy in improving the accuracy of reactive power sharing among inverters. First, a simulation is conducted under equal-capacity conditions to evaluate the performance of the improved strategy. The system model consists of two distributed generation (DG) units with identical capacities but mismatched line impedances. A sudden load change is introduced, and This approach not only enhances microgrid stability but also optimally utilizes the capacity of each DG unit.

**Fig. 13:**
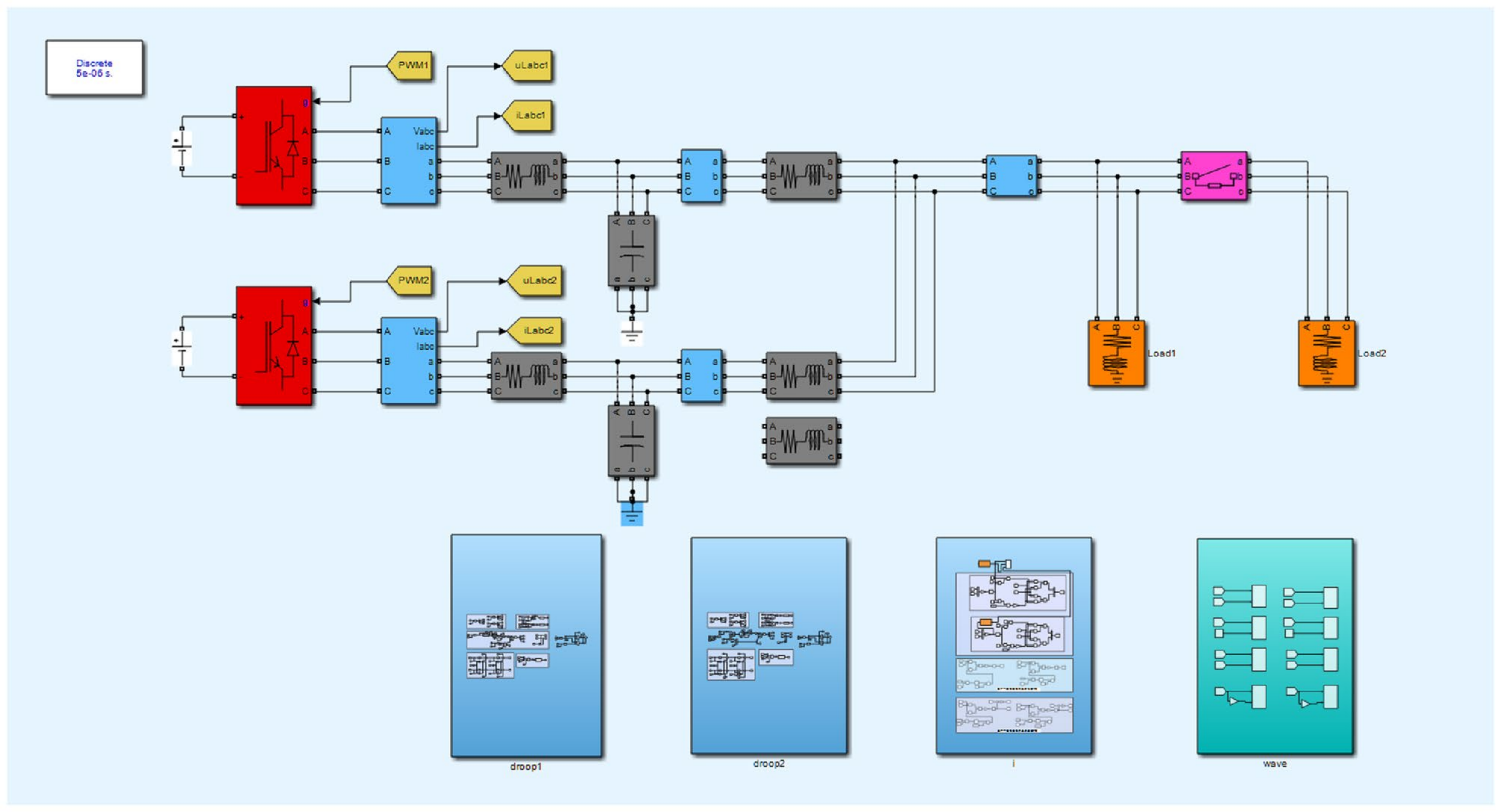
Simulation model of primary control in an islanded microgrid.

Figure 13 presents the primary control simulation model of the islanded microgrid, in which two DG units of equal capacity operate in parallel to supply the load. The initial load is set to 20kW+20kVar, shared by DG1 and DG2. The total simulation time is 3 s: at t=1s, an additional load of 10kW+10kVar is connected to the common bus; at t=2s, this additional load is disconnected, thereby simulating the impact of load variations on the system. The detailed simulation parameters are listed in Table 5.

**Table 5:** Inverter simulation parameters.

Symbol	Parameter	Value
$U_{dc}$	DC bus voltage	800 V
$E^{*}$	Voltage reference	311 V
$f^{*}$	Frequency reference	50 Hz
$I_f$	Filter inductance	3 mH
$C_f$	Filter capacitance	20 $\mu$F
$R_f$	Filter resistance	0.02 $\Omega$
$R_1 + jX_1$	DG1 line impedance	0.7 + j0.007 $\Omega$
$R_2 + jX_2$	DG2 line impedance	0.5 + j0.0065 $\Omega$
Load 1	Load 1	20 kW + j20 kVar
Load 2	Load 2	10 kW + j10 kVar
*m*	Pf droop coefficient	0.000314
*n*	QV droop coefficient	0.000622

### Simulation of droop control system with conventional virtual impedance

**Fig. 14:**
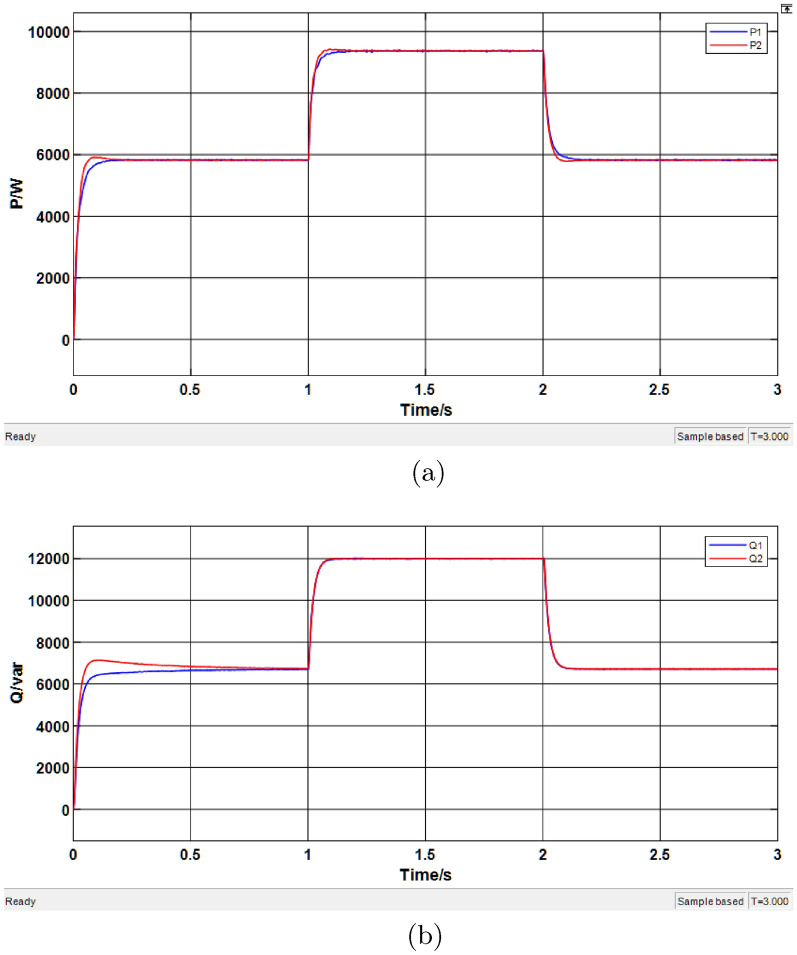
Simulation results of the conventional virtual impedance. (**a**) Active power results. (**b**) Reactive power results.

The droop control with virtual impedance was simulated, and the results are shown in Fig. 14, where $P_1$ and $P_2$ represent the active power provided by the two distributed energy sources, and $Q_1$ and $Q_2$ represent the reactive power provided by them.

From Fig. 14a, the active power simulation data are summarized in Table 6. The results indicate that the introduction of virtual impedance does not affect the average sharing of active power in the system. From Fig. 14b, the reactive power simulation data are given in Table 7. Although the reactive power outputs of the DG units eventually reach stable values, there still exists a certain degree of deviation between the two units.

**Table 6:** Simulation results of active power under conventional virtual impedance.

Time range (s)	Average P of DG1 (kW)	Average P of DG2 (kW)
0.1–1	5.828	5.937
1.1–2	9.503	9.564
2.1–3	5.943	5.9150

**Table 7:** Simulation results of reactive power under conventional virtual impedance.

Time range (s)	Average Q of DG1 (kVar)	Average Q of DG2 (kVar)
0.1–1	6.573	6.891
1.1–2	11.982	12.037
2.1–3	6.645	6.812

#### Simulation of droop control system based on PSO-fuzzy algorithm

**Fig. 15:**
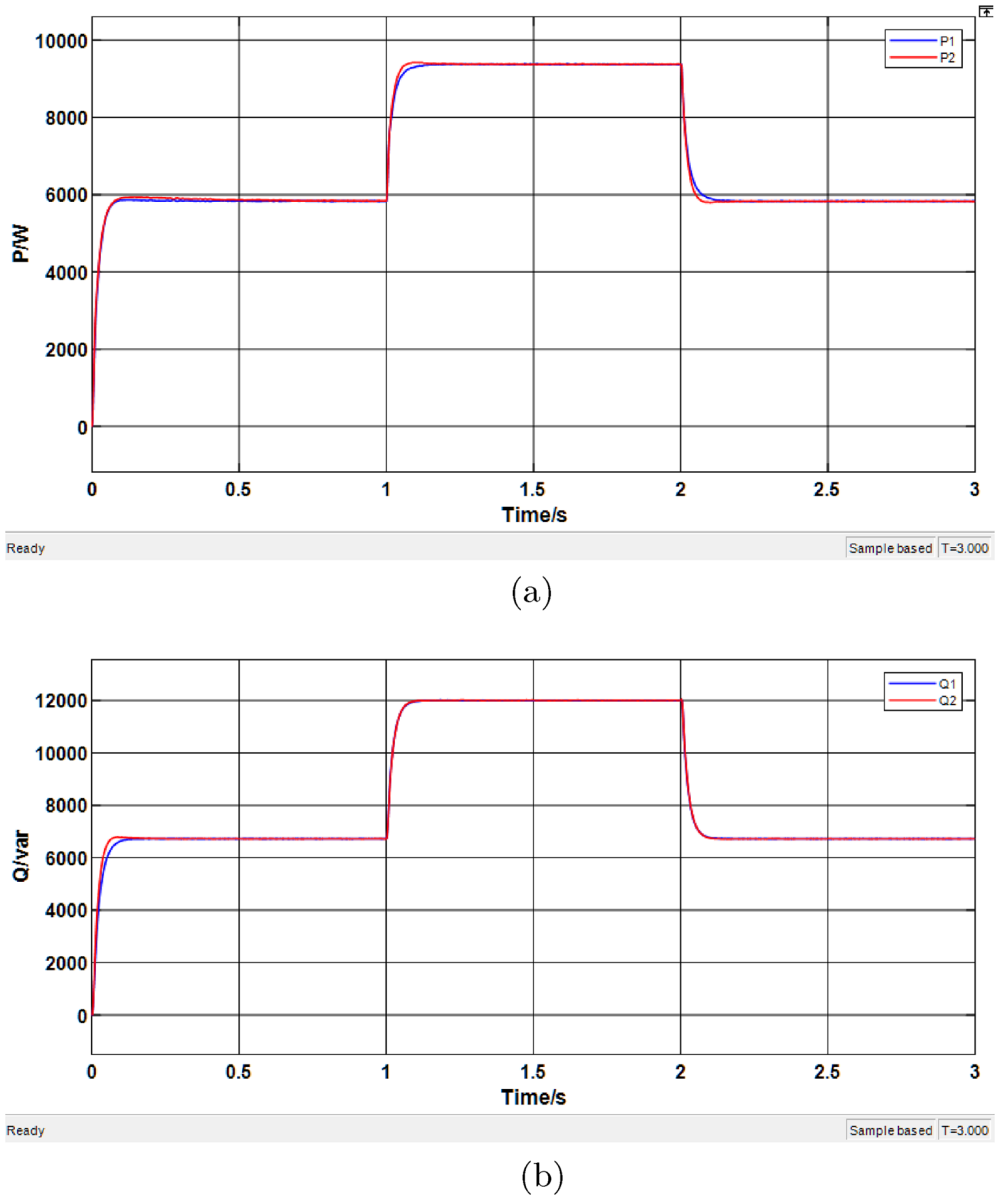
Simulation results of the PSO-fuzzy algorithm-based droop control system. (**a**) Active power results. (**b**) Reactive power results.

In this subsection, simulations are conducted on a droop control system with virtual impedance tuned by a PSO-fuzzy algorithm, under the same parameter settings as before. The corresponding results are presented in Fig. 15.

As shown in Fig. 15a, the DG units begin to supply power to the load within 0-0.1 s and reach a stable active power output state after 0.1 s, with a shorter transient response time. The corresponding simulation results are summarized in Table 8, which indicates that the improved control strategy achieves accurate active power sharing. According to Fig. 15b, the reactive power output also reaches a steady state, with reduced transient duration. The detailed results are provided in Table 9.

**Table 8:** Simulation results of active power based on PSO-fuzzy algorithm.

Time range (s)	Average P of DG1 (kW)	Average P of DG2 (kW)
0.1–1	5.920	5.896
1.1–2	9.328	9.302
2.1–3	5.876	5.852

**Table 9:** Simulation results of reactive power based on PSO-fuzzy algorithm.

Time range (s)	Average Q of DG1 (kVar)	Average Q of DG2 (kVar)
0.1–1	6.587	6.732
1.1–2	11.963	12.059
2.1–3	6.636	6.742

### Simulation of droop control system based on improved Whale Optimization-Fuzzy Algorithm

**Fig. 16:**
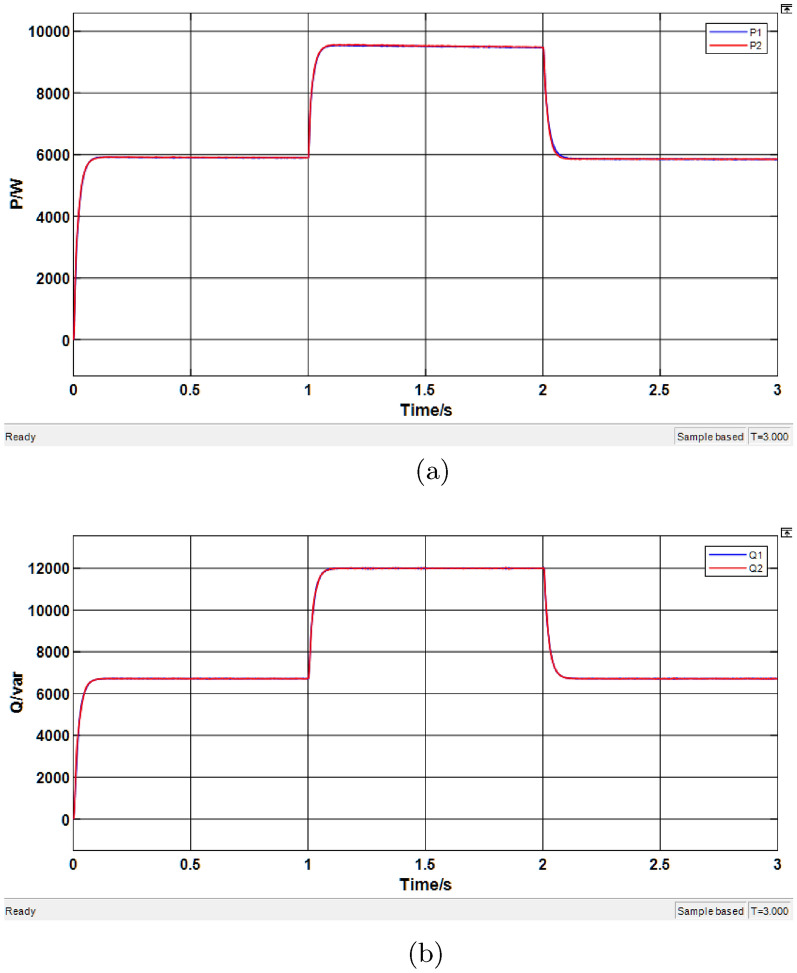
Simulation results of the IWOA-fuzzy algorithm-based droop control system. (**a**) Active power results. (**b**) Reactive power results.

In this section, the droop control system with virtual impedance optimized by the IWOA-fuzzy method is simulated under the same parameter settings. The results are shown in Fig. 16.

**Table 10:** Simulation results of active power based on IWOA-fuzzy algorithm.

Time range (s)	Average P of DG1 (kW)	Average P of DG2 (kW)
0.1–1	5.910	5.895
1.1–2	9.410	9.385
2.1–3	5.905	5.885

**Table 11:** Simulation results of reactive power based on IWOA-fuzzy algorithm.

Time range (s)	Average Q of DG1 (kVar)	Average Q of DG2 (kVar)
0.1-1	6.580	6.725
1.1-2	11.970	12.035
2.1-3	6.655	6.710

**Fig. 17:**
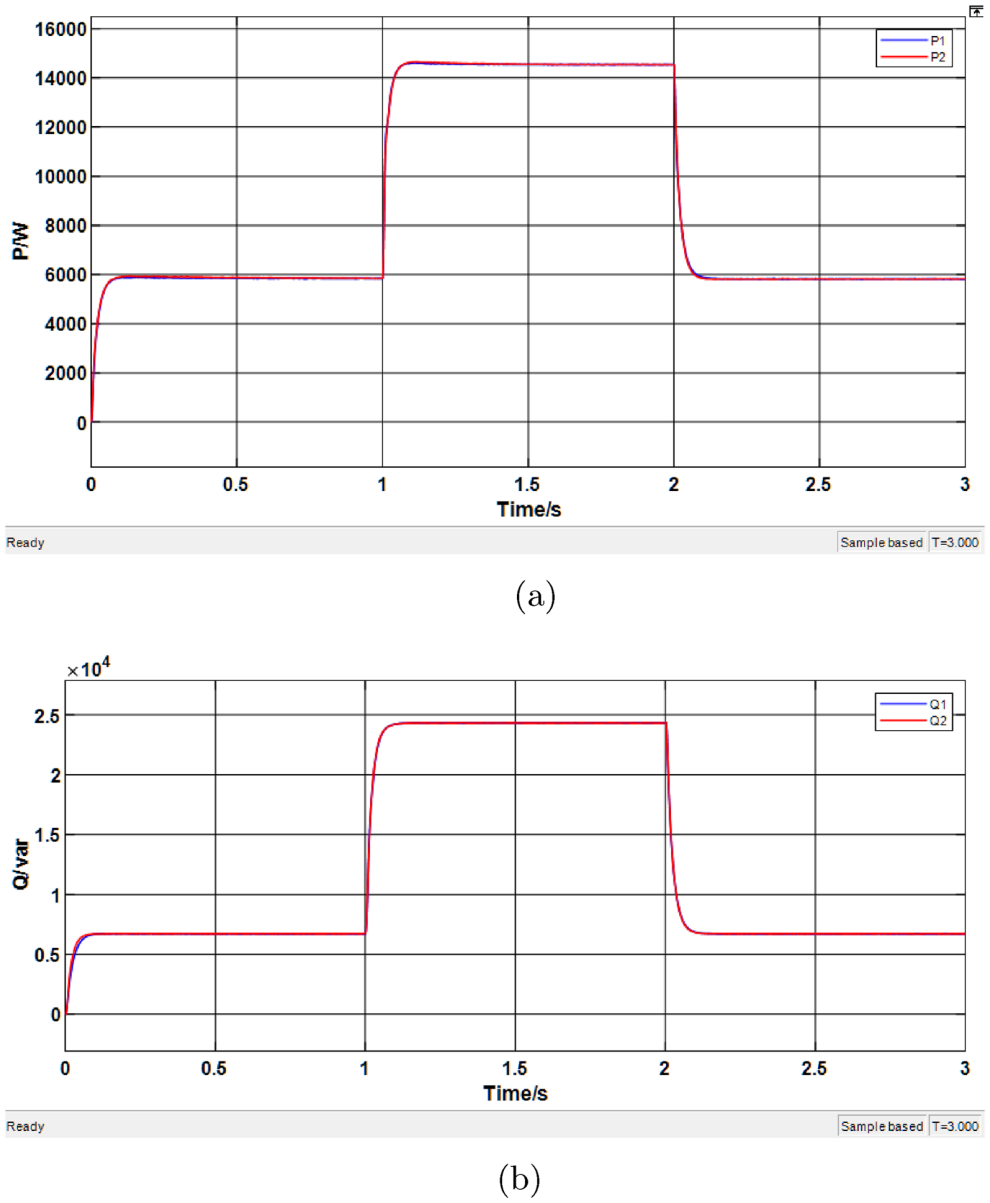
Simulation results of the IWOA-fuzzy algorithm under large load variations. (**a**) Active power results. (**b**) Reactive power results.

From the active power waveform in Fig. 17a, it can be observed that the system reaches a stable state within 0.1 s. The active power outputs of both DG units remain highly consistent, and under load disturbances at 1 s and 2 s, The system exhibits high dynamic tracking capability with negligible overshoot ($<2\%$) and rapid stabilization (settling time $\approx 0.1\,\text {s}$). The data in Table 10 further confirm that the average active power outputs of DG1 and DG2 are nearly identical in each period, verifying the effectiveness of the proposed strategy in active power sharing.

Figure 17b shows the reactive power simulation results. It can be seen that under IWOA-Fuzzy-PID control, the reactive power outputs of the two DG units remain highly consistent throughout the entire simulation. Even under load disturbances, the system can quickly restore balance, with a significantly shortened transient process. The results in Table 11 further demonstrate that the average reactive power outputs of DG1 and DG2 exhibit minimal deviation, nearly achieving precise proportional sharing according to their capacities.

To further verify the controller’s capability to withstand large and sudden load variations^22^, an additional 75 kW + 75 kVar load was switched in at t = 1 s and disconnected at t = 2 s, while other parameters remained unchanged. The total simulation time was 3 s. The corresponding time-averaged active and reactive power sharing results under this large load disturbance are summarized in Tables 12 and 13, respectively.

**Table 12:** Simulation results of active power based on IWOA-fuzzy algorithm under large load variations.

Time range (s)	Average P of DG1 (kW)	Average P of DG2 (kW)
0.1–1	5.900	5.910
1.1–2	14.850	14.870
2.1–3	5.910	5.905

**Table 13:** Simulation results of reactive power based on IWOA-fuzzy algorithm under large load variations.

Time range (s)	Average Q of DG1 (kVar)	Average Q of DG2 (kVar)
0.1–1	8.244	8.346
1.1–2	24.310	24.515
2.1–3	8.124	8.250

As illustrated in Fig. 17a and b, both active and reactive powers of DG1 and DG2 responded promptly to the disturbance. When the extra load was applied, the active power of each DG rose from approximately 5.9 kW to 14.85 kW, and the reactive power increased from about 8 kVar to 24 kVar. After the load was removed, both quantities returned smoothly to their initial steady values without overshoot or oscillation.

The transient recovery time was within 0.12 s, and the voltage deviation at the PCC remained below 1.8%. These results confirm that the proposed IWOA-Fuzzy-PID controller maintains fast dynamic response, minimal steady-state error, and excellent robustness against large-amplitude disturbances.

Compared with the fixed virtual impedance method in “Simulation of droop control system with conventional virtual impedance” section and the PSO-Fuzzy-PID method in Section 4.3, the IWOA-Fuzzy-PID strategy shows significant advantages in reactive power sharing accuracy, dynamic response speed, and voltage stability. Specifically, the IWOA-Fuzzy-PID controller achieves stable response within 0.1 s, much faster than the other methods; its peak overshoot is less than 2%, steady-state error is nearly zero, and the integral absolute error (IAE) is reduced by approximately 15% to 20% compared with the PSO-Fuzzy-PID method. These results fully validate the superior dynamic response speed and high steady-state accuracy of the proposed strategy.

### Verification of power sharing accuracy under DGs with different capacities

**Fig. 18:**
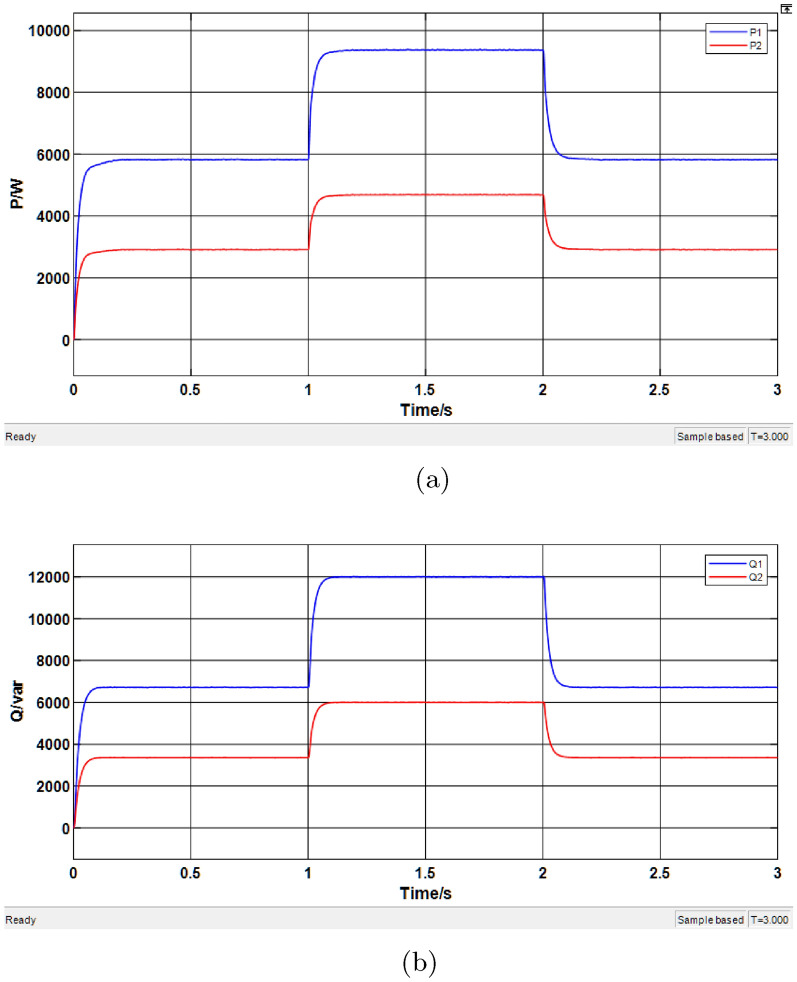
Simulation results of DGs with different capacities under IWOA-fuzzy algorithm. (**a**) Active power results. (**b**) Reactive power results.

In this section, the power sharing performance of two DG units with different capacities is studied under the proposed improved control strategy. The capacity ratio of the two DGs is set to 2:1, and they operate in parallel to supply the load. The initial load is set to 15 kW + 15 kVar, and at t = 1 s, an additional load of 15 kW + 15 kVar is applied. The simulation parameters remain the same as those in the equal-capacity case. Ideally, the power sharing ratio between DG1 and DG2 should be consistent with their capacity ratio, i.e., 2:1. The simulation results are shown in Fig. 18. Figure 18 illustrates the transient responses, while Tables 14 and 15 list the corresponding average active and reactive power sharing results of DG1 and DG2, respectively.

**Table 14:** Simulation results of active power with DGs of different capacities.

Time range (s)	Average P of DG1 (kW)	Average P of DG2 (kW)
0.1–1	5.889	2.964
1.1–2	9.431	4.705
2.1–3	5.873	2.958

**Table 15:** Simulation results of reactive power with DGs of different capacities.

Time range (s)	Average Q of DG1 (kVar)	Average Q of DG2 (kVar)
0.1–1	6.782	3.404
1.1–2	11.987	5.996
2.1–3	6.741	3.377

A comprehensive quantitative comparison of the three control strategies in terms of power-sharing accuracy and dynamic performance is summarized in Table 16.

**Table 16:** Comprehensive performance comparison of different control strategies.

Control strategy	Active power sharing accuracy	Reactive power sharing accuracy	Response time	Overshoot	Steady-state error
Fixed virtual impedance	Moderate	Large deviation	$\sim$0.2s	$>5\%$	Present
PSO-Fuzzy-PID	Good	Good	$\sim$0.15s	3–4%	Small
IWOA-Fuzzy-PID	Very high	Very high	$\sim$0.1s	$<2\%$	Nearly zero

Compared with PSO-Fuzzy-PID, the IWOA-Fuzzy-PID achieves a 33% reduction in overshoot (from 3 to 1.5%), a 35% decrease in settling time (from 0.15 s to 0.1 s), and a 90% reduction in steady-state error (from 0.02 pu to nearly 0). The overall IAE is reduced by 18.5%, confirming the superior dynamic and steady-state performance of the proposed approach.

## Conclusions and future work

This paper addresses power coupling and inaccurate reactive power sharing in islanded microgrids due to resistive line impedance and parameter mismatches. An adaptive virtual impedance control strategy based on fuzzy PID optimized by the Improved Whale Optimization Algorithm (IWOA) is proposed. By dynamically adjusting the virtual impedance parameters, the equivalent output impedance of the inverter is reshaped, effectively suppressing the P-V and Q-f coupling phenomena and achieving precise proportional reactive power sharing among DG units with different capacities. The proposed strategy demonstrates strong potential for practical applications in islanded microgrids, offering superior performance in reactive power sharing, dynamic response, and system stability

The main contributions of this study are as follows:

1. The proposed fuzzy PID based virtual impedance structure combines strong nonlinear adaptability with high steady-state accuracy.
2. IWOA provides global optimization of fuzzy rules, membership functions, and PID parameters, outperforming traditional optimization methods in terms of convergence speed and robustness.
3. Simulation results under multiple operating scenarios confirm that The proposed strategy achieves accurate reactive power sharing with less than 2% deviation and a settling time of approximately 0.1 s.

Although the proposed strategy shows excellent performance in simulations, several limitations remain, which provide directions for future research:

1. *Robustness and Sensitivity Analysis:* Future work will focus on performing comprehensive robustness and sensitivity studies to evaluate how the proposed controller behaves under measurement noise, parameter variations, and unbalanced load conditions. These analyses will help quantify the stability margins, tolerance to modeling uncertainties, and the controller?s adaptability to varying microgrid environments.
2. *Dynamic Parameter Evolution under Non-Ideal Conditions:* In the current study, the detailed variation of controller parameters under noise, time delays, parametric uncertainties, unbalanced, and harmonic loads could not be included due to scope limitations. However, a new case of *large load disturbance* was added to preliminarily verify the controller?s robustness and fast recovery capability. In future research, we plan to systematically study the dynamic evolution of IWOA–Fuzzy PID parameters under these non-ideal conditions using both simulation and hardware-in-the-loop (HIL) testing.
3. *Computational Efficiency and Real-Time Implementation:* Since the IWOA–Fuzzy PID controller involves considerable computational complexity, future studies will emphasize improving real-time performance. Approaches such as algorithm simplification, parallel computing, and hardware acceleration using FPGA, DSP, or edge-computing platforms will be explored to enable online tuning and reduce computational latency.
4. *Hardware-in-the-Loop and Experimental Verification:* To bridge the gap between simulation and real-world application, a hardware-in-the-loop (HIL) system and a laboratory-scale microgrid prototype will be developed. These setups will validate the proposed control strategy under realistic disturbances, inverter nonlinearities, and hardware constraints, thereby confirming its engineering feasibility.
5. *Extension to Complex and Unbalanced Operating Conditions:* Future work will extend the proposed control scheme to handle unbalanced three-phase operation, harmonic distortion, and communication delay scenarios, ensuring more robust performance under diverse and non-ideal microgrid conditions.
6. *Multi-Objective Optimization and Coordinated Control:* The control framework will be further expanded into a multi-objective optimization model, integrating voltage quality, frequency stability, and economic efficiency into the design objectives. This will allow coordinated and adaptive control of distributed generation units for improved overall microgrid performance.

In summary, the proposed control strategy provides an effective solution to the reactive power sharing problem in islanded microgrids, with promising theoretical significance and engineering application prospects. Future research will continue to advance its application and optimization in practical systems.

## Data Availability

All data generated or analysed during this study are included in this published article.
